# Isolation and whole-genome characterization of an emerging G9P[23] porcine rotavirus in Central China

**DOI:** 10.3389/fvets.2025.1708098

**Published:** 2025-12-19

**Authors:** Chaoliang Leng, Jiabao Wang, Jiajing Song, Jiajia Cao, Mengtian Jia, Tianyu Jia, Linyue Xiao, Yingying Zhao, Yu Zhang, Jiayin Zhang, Hongyue Zhai, Na Li, Hongfei Shi, Dandan Li, Yunchao Kan, Lunguang Yao, Zhi-Jun Tian

**Affiliations:** 1 Henan Provincial Engineering and Technology Center of Animal Disease Diagnosis and Integrated Control, Henan Key Laboratory of Insect Biology, Henan Province Engineering Research Center of Insect Bioreactor, China-UK International Joint Laboratory for Insect Biology of Henan Province, Nanyang Normal University, Nanyang, China; 2 State Key Laboratory for Animal Disease Control and Prevention, Harbin Veterinary Research Institute, Chinese Academy of Agricultural Sciences, Harbin, China

**Keywords:** evolutionary characterization, G9P[23], isolation, porcine rotavirus, reassortment, whole-genome sequencing

## Abstract

Rotavirus (RV), a significant enteric pathogen, causes diarrheal disease in both nursing piglets and infants. To characterize the genomic features and evolutionary patterns of a porcine rotavirus (PoRV) strain, we isolated viral strains from RT-qPCR confirmed PoRV-positive diarrheic piglet specimens through MA104 cell culture inoculation. Viral isolates were definitively characterized through RT-PCR amplification, indirect immunofluorescence assay (IFA), and transmission electron microscopy (TEM), followed by comprehensive whole-genome sequencing and phylogenetic analysis. The results demonstrated successful isolation of a G9P[23] genotype PoRV, designated HN240916. Whole-genome analysis revealed that the genotype constellation was G9-P[23]-I5-R1-C1-M1-A8-N1-T1-E1-H1. Genomic analysis revealed that the NSP3 and NSP5 genes of the HN240916 strain cluster monophyletically with human RVs, while all remaining genes exhibit close phylogenetic affinity to PoRV lineages. The genotype constellation provides genomic evidence of a past interspecies reassortment event between porcine and human RVs. This study expands PoRV molecular epidemiology resources and enables genotype-based prevention strategies.

## 1 Introduction

Rotavirus (RV) constitutes a leading etiological agent of viral gastroenteritis in human infants and young animals. Its zoonotic potential poses significant risks to pediatric health and imposes considerable economic losses in livestock industries, particularly swine and cattle production (1–4). Porcine rotavirus (PoRV) infection primarily induces severe diarrhea in 1–4-week-old piglets (5). Notably, PoRV frequently co-infects with other critical viral pathogens, such as porcine epidemic diarrhea virus (PEDV) and transmissible gastroenteritis virus (TGEV), leading to synergistically exacerbated disease severity (6, 7).

According to the latest classification of International Committee on Taxonomy of Viruses (ICTV), RV belongs to the family *Sedoreoviridae* (8). The genome consists of 11 segments of RNA encoding six structural viral proteins (VP1 to VP4, VP6, and VP7) and five or six nonstructural proteins (NSP1 to NSP5/6), and the total genome contains approximately 18,522 nucleotides (9, 10). According to the nucleotide differences of the inner capsid protein (VP6), RVs are currently classified into 9 species: RV alphagastroenteriditis (RVA), RV betagastroenteriditis (RVB), RV tritogastroenteritidis (RVC), RV deltagastroenteriditis (RVD), RV phigastroenteritidis (RVF), RV gammagastroenteritidis (RVG), RV aspergastroenteritidis (RVH), RV iotagastroenteriditis (RVI), and RV jotagastroenteritidis (RVJ) (8). The serotype and genotype specificity of RV is determined by its bilayer capsid proteins, VP7 (glycoprotein) and VP4 (protease-sensitive protein). The viruses are classified into two major groups, G-type (VP7 glycoprotein) and P-type (VP4 protease-sensitive), on the basis of antigenic differences between these two antigens (11). Among species A, B, C, and H RVs that have been identified in swine, RVA is considered the most prevalent and pathogenic (12, 13). To date, 27 different G genotypes and 37 different P genotypes of species A RVs have been identified in humans and animals (14, 15). The predominant genotypes of PoRV in China at present are G5 and G9 types, in addition to P[7], P[13], P[19] and P[23]. A hallmark characteristic of PoRV strains is their pronounced propensity to undergo genetic reassortment, occurring readily in natural settings or under experimental conditions. Research has indicated the potential for genetic reassortment to occur between PoRV and human RV strains. Such reassortment events have been observed to be associated with the development of human diarrhea (16). In recent years, PoRV G9 type has been recognized as an emerging genotype that is spreading globally and that continues to undergo genetic reassortment between humans and animals (17–20). Recent studies have reported porcine-human reassortment G9P[23] strains in China (21, 22), highlighting its widespread circulation. However, knowledge gaps remain regarding the geographic distribution, segmental reassortment patterns, and evolutionary dynamics of these strains, particularly in underrepresented provinces.

In this experiment, one PoRV strain, designated as HN240916, was isolated from the intestinal samples of diarrheic piglets in a large-scale pig farm in Henan Province, and classified according to the most recent method released by the Rotavirus Classification Working Group (RCWG). Whole-genome sequences of HN240916 were deposited in GenBank for comparative analysis via NCBI BLAST. Phylogenetic trees were constructed based on all the 11 gene sequences, with a comparative analysis of amino acid variations of the VP7 and VP4 proteins.

## 2 Materials and methods

### 2.1 Sampling and examining

During an outbreak investigation at a commercial swine farm in Henan Province, central China, approximately 15-day-old piglets presented with watery diarrhea. Intestinal tissues were collected for pathogen diagnostics and virus isolation. Initial screening employed quantitative real-time PCR (qPCR) using commercial kits (Guanmu, Changsha, China) targeting PEDV, TGEV, PoRV, and porcine deltacoronavirus (PDCoV). PoRV-positive samples were subjected to Sanger sequencing with VP7-targeting primers (Sense: 5′-ATGTATGGTATTGAATATACCAC-3′; Antisense: 5′-AACTTGCCACCATTTTTTCC-3′) for validation and genotyping.

### 2.2 Virus isolation

PoRV isolation was performed using MA104 cells according to a previously described method with minor modifications (23). Intestinal contents were homogenized in serum-free Dulbecco’s Modified Eagle Medium (DMEM) (Solarbio, Beijing, China) supplemented with 1% penicillin–streptomycin (Solarbio, Beijing, China) by liquid nitrogen grinding. The homogenate was filtered through a 0.22-μm pore filter, added the trypsin with a final concentration of 30 μg/mL, and incubated at 37 °C for 1 h. When the cells in six-well plates reached 80–90% confluency, culture medium was aspirated and monolayers were washed three times with pre-equilibrated serum-free DMEM, followed by replenishment with 2 mL of fresh medium. A total of 500 μL of homogenate was added to the six-well plate. After 2 h of incubation at 37 °C, the homogenate was removed and replaced with maintenance medium containing trypsin at a final concentration of 5 μg/mL. The cells were monitored daily for cytopathic effects (CPEs), characterized by cell elongation, aggregation, and detachment. The infected cells were harvested when approximately 80% of the cell monolayer exhibited characteristic CPEs. The cells were centrifuged and supernatants were subjected to passage after three freeze–thaw cycles at −80 °C. The fifth viral harvests were characterized and purified using the limited-dilution method.

### 2.3 Indirect immunofluorescence assay (IFA)

IFA was conducted as previously described (24). Viral antigens were prepared by inoculating MA104 cells with the newly isolated fifth-passage (P5) PoRV strain. A specific monoclonal antibody against the RV VP6 protein (diluted 1:200) (Medix Biochemica, Shanghai, China) and goat anti-mouse IgG antibody conjugated with FITC (diluted 1:500) (Solarbio, Beijing, China) were used as the primary and secondary antibodies, respectively.

### 2.4 Sequence analysis

The complete genome of the isolated P5 PoRV strain was sequenced using the high-throughput sequencing (HTS) platform at South China Agricultural University as described previously (25). Briefly, total RNA was extracted from the fifth-generation strain using the TRIzol reagent (Solarbio, Beijing, China). During library construction, ribosomal RNA was removed using the Ribo-MagOff rRNA depletion kit (Vazyme, Nanjing, China), and 150-bp paired-end libraries were prepared using the VAHTS Universal V8 RNA-seq Library Prep Kit (Vazyme, Nanjing, China). The qualified libraries were sequenced on an Illumina NovaSeq 6000 platform (Illumina, USA). After sequencing, raw reads were subjected to quality control using *fastp* to remove adaptor sequences and low-quality reads. The filtered reads were then assembled *de novo* using *SPAdes* and *SOAPdenovo* assemblers to reconstruct the viral genome. The contigs with a length of ≥300 bp were retained for further analysis and depth statistics. The resulting assembled sequences were compared with reference PoRV genomes available in GenBank to confirm genome completeness and accuracy.

The HTS results were uploaded to NCBI for BLAST comparison. Viral genotype determination was performed using the automated genotyping tool within the Virus Pathogen Resource (ViPR, https://www.viprbrc.org/). According to the standards of the RCWG, PoRV genotypes are considered identical when nucleotide sequence identities exceed established thresholds across all 11 genomic segments. To ensure accurate classification, all 11 genomic RNA segments of the isolated P5 PoRV strain were further genotyped following the criteria outlined by Matthijnssens et al. (26). Phylogenetic analyses were conducted using the maximum-likelihood (ML) method implemented in *MEGA 11* software, employing the GTR substitution model with 1,000 bootstrap replicates, following the approach of Matthijnssens et al. (27). Representative reference human and porcine RV sequences were retrieved from GenBank for comparison. To investigate the genetic and evolutionary relationships of the PoRV strain, phylogenetic trees were constructed based on all the 11 gene sequences obtained in this study, together with reference sequences from GenBank. Additionally, amino acid sequences of VP7 and VP4 were analyzed using the pairwise alignment algorithm in *MegAlign* to evaluate the genetic variation of the PoRV strain.

## 3 Results

### 3.1 Molecular identification of PoRV

qPCR analysis showed exclusive positivity for PoRV, with PEDV, TGEV, and PDCoV testing negative. Purified amplicons were commercially sequenced using VP7-specific primers (General Biosystems, Anhui, China), and subsequent GenBank BLAST alignment confirmed sequence identity with PoRV, definitively verifying PoRV-specific nucleic acids in diarrheic specimens and the genotype of its VP7 gene as the G9 subtype.

### 3.2 Virus isolation and identification

**Figure 1:**
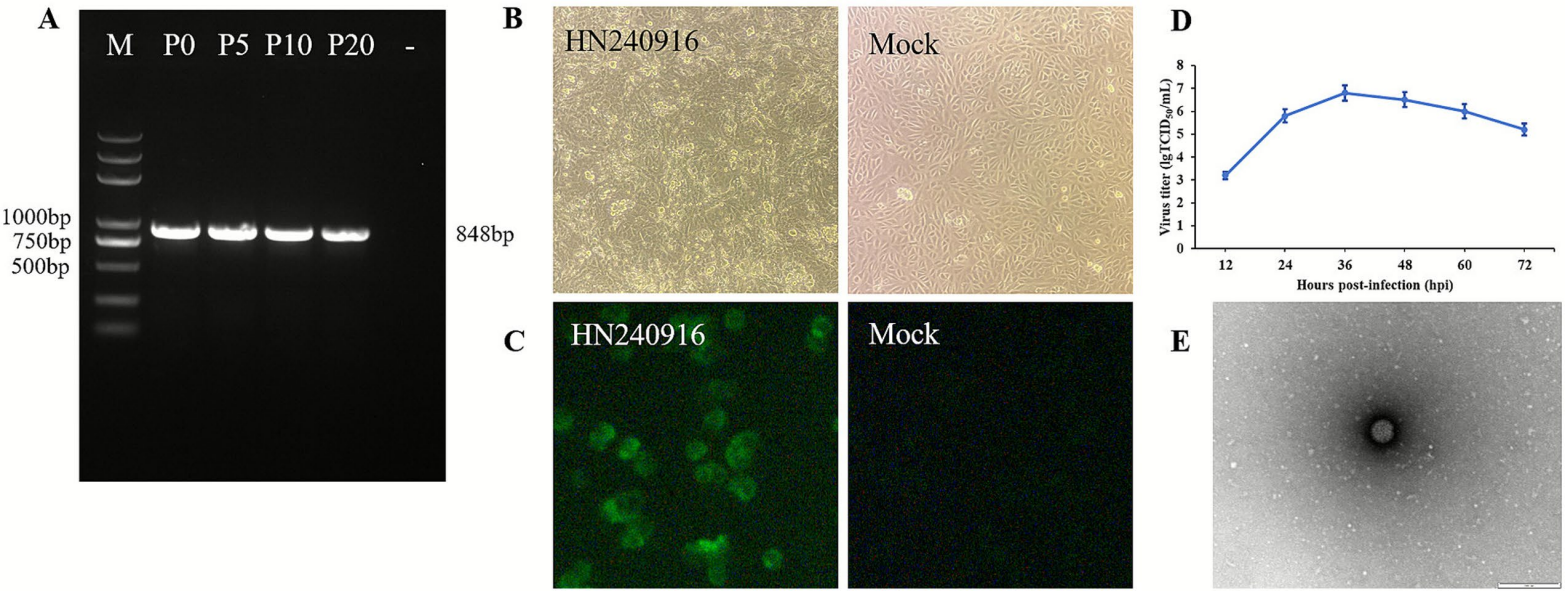
Virus isolation, identification, and growth curves of HN240916. **(A)** RT-PCR products using the VP7-targeting primers for 0, 5, 10, and 20 successive generations of the HN240916 strain. **(B)** CPE infected with HN240916-P5 strain in MA104 cells. Magnification, 100×. **(C)** VP6 protein expression in HN240916-P5-infected MA104 cells analyzed by IFA. Magnification, 400×. **(D)** Growth kinetics of HN240916-P5 in MA104 cells. Data were presented as mean ± SD of triplicates. **(E)** Electron microscopic images of purified HN240916-P5 virions. Scale bars, 100 nm.

We attempted to isolate PoRV from the PCR-positive diarrhea samples that did not contain other pathogens. Following three serial blind passages in MA104 cells, CPEs manifested at 24 h post-inoculation (hpi) and progressed to complete CPE by 48 hpi. We referred to the strain as HN240916. To verify whether the strain could maintain its viability and replication capacity in MA104 cells, it was continuously propagated for 20 generations. Subsequently, the VP7 genes of the P0, P5, P10, and P20 generations of the virus were identified as positive (Figure 1A). The typical CPEs of HN240916-P5 were characterized by initial cell rounding and elongation, followed by loss of cellular boundaries and monolayer detachment subsequently (Figure 1B). In contrast, mock MA104 cells exhibited no CPEs (Figure 1B). IFA with anti-VP6 monoclonal antibody revealed distinct cytoplasmic green fluorescence in the HN240916-P5-infected MA104 cells at 24 hpi, whereas the mock cells exhibited no specific signal (Figure 1C). The growth kinetics analysis revealed that HN240916-P5 replicated effectively in MA104 cells. At 36 hpi, the viral infection titer peaked at 10^6.8^ TCID_50_/mL (Figure 1D). Additionally, the typical particles of the HN240916-P5 strain, which were separated from the infected cells, were examined by transmission electron microscopy (TEM). Negative staining under the electron microscope revealed particles with a wheel-like feature, closely resembling spheres. These particles had an approximate diameter of 70 nm (Figure 1E).

### 3.3 Genome sequences of HN240916

After HTS performed using the Illumina Novaseq 6000 platform, the complete sequences of 11 gene segments of the HN240916-P5 strain were successfully identified. According to the latest classification and naming system established by the RCWG, the nucleotide identity cutoff values for 11 gene segments of RV Group A are as follows: 80% (G), 80% (P), 85% (I), 83% (R), 84% (C), 81% (M), 79% (A), 85% (N), 85% (T), 85% (E), and 91% (H) (26). Sequence alignment, nucleotide BLAST analysis, and nucleotide sequence identity analysis all confirmed that HN240916-P5 strain was identified as G9-[P23]-I5-R1-C1-M1-A8-N1-T1-E1-H1 genotypes (Table 1). The sequences of all 11 gene segments were uploaded to GenBank with accession numbers PX214352 to PX214362. Homology analysis was performed with closely related RV reference strains. Multiple gene segments, including VP1, VP2, VP3, VP4, VP6, VP7, NSP1, NSP2, and NSP4 exhibited high homology with PoRV strains. Additionally, NSP3 and NSP5 showed higher homology with human RV strains (Table 1).

**Table 1:** The highest nucleotide sequence identity between HN240916 and other known RV strains.

Gene	Virus with the highest identity	Country	Year	Identity (%)	Genotype	Species	Accession No.
VP1	RVA/Pig/China/SC11/2017/G9P[23]	China	2017	95.78	R1	Porcine	MH624173.1
VP2	RVA/Pig-wt/CHN/923E/2021/G9P[23]	China	2021	97.64	C1	Porcine	PQ141607.1
VP3	RVA/Pig-wt/VNM/14225_44/VP3	Vietnam	2012	97.61	M1	Porcine	KX363369.1
VP4	RVA/pig/CHN/HeN/10437/2023/G9P23I5	China	2023	98.63	P23	Porcine	PV421675.1
VP6	RVA/Pig/HuNan-2RV/2023	China	2023	98.58	I5	Porcine	OR094881.1
VP7	RVA/pig/CHN/SC/06041/2023/G9P23I5	China	2023	97.45	G9	Porcine	PV421418.1
NSP1	RVA/Pig/China/SC11/2017/G9P[23]	China	2017	96.86	A8	Porcine	MH624168.1
NSP2	RVA/Pig/CHN/JSJR2023	China	2023	98.22	N1	Porcine	PP100157.1
NSP3	RVA/Human/LL4260/China/T1	China	2001	96.68	T1	Human	KC149929.1
NSP4	RVA/pig/China/NMTL/2008/G9P[23]	China	2008	98.30	E1	Porcine	JF781167.1
NSP5	RVA/Human-wt/CHN/E931/2008/G4P[6]	China	2008	98.65	H1	Human	KF726043.1

### 3.4 Phylogenetic analysis of the full-length gene

**Figure 2:**
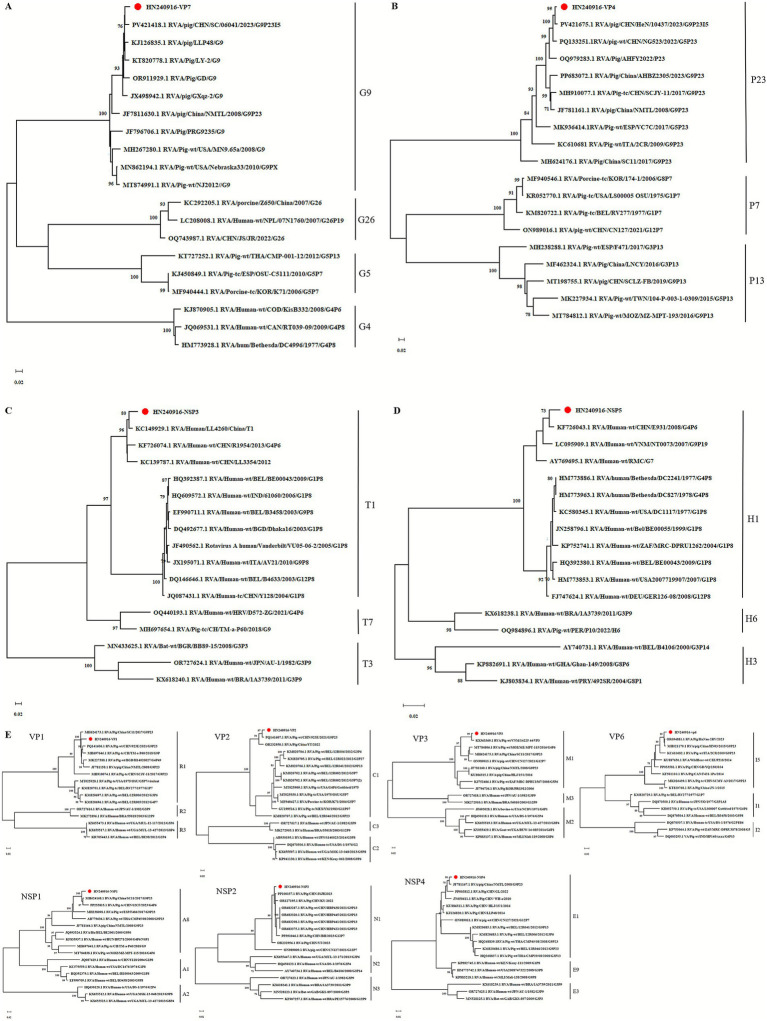
Phylogenetic trees based on VP7 **(A)**, VP4 **(B)**, NSP3 **(C)**, NSP5 **(D)**, VP1–VP3, VP6, NSP1–NSP2, and NSP4 **(E)**. 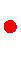 Display HN240916-P5 strain sequence.

To further investigate the genetic relatedness of HN240916-P5 among other G9 and non-G9 strains, as well as among P23 and non-P23 strains, nucleotide similarity and phylogenetic analyses were conducted using their corresponding genomic sequences. The VP7 gene comprises 981 nucleotides, encoding 326 amino acids. The nucleotide identity ranged from 89.8 to 97.5%, and the amino acid identity ranged from 93.0 to 99.1% among the selected G9 genotypes from GenBank. A phylogenetic tree was constructed using the full-length encoding VP7 sequence together with representative G-genotypes (G4, G5, G9, and G26) sequences obtained from GenBank (Figure 2A). The VP7 of the HN240916-P5 strain clustered within the G9 genotype, a group primarily composed of porcine-origin strains. The sequence most closely related to the HN240916-P5 VP7 gene was identified as originating from the PoRV strain, RVA/pig/CHN/SC/06041/2023/G9P[23]I[5] (PV421418.1), which was obtained in China in 2023. The VP4 gene comprises 2,331 nucleotides, encoding a protein of 776 amino acids. The nucleotide identity ranged from 88.1 to 98.6%, and amino acid identity ranged from 94.9 to 99.7% for the chosen P[23] genotypes in GenBank. A phylogenetic tree was built based on the full-length VP4 sequence of HN240916-P5 strain along with P genotypes (P[6], P[7], P[13], and P[23]) sequences selected from GenBank (Figure 2B). The VP4 of the HN240916-P5 strain clustered within the P[23] genotype, which were composed of the porcine-origin isolates from China and other countries. The VP4 of the HN240916-P5 showed the closest relationship to RVA/pig/CHN/HeN/10437/2023/G9P23I5 (PV421675.1), also isolated from China.

Genomic analysis revealed that the NSP3 gene contains 933 nucleotides, encoding 310 amino acids, whereas the NSP5 gene comprises 594 nucleotides, encoding a 197 amino acids protein. Compared with the selected T1 genotype strains, the NSP3 of the HN240916-P5 strain exhibited nucleotide and amino acid identities ranging from 85.1 to 96.7% and 91.6 to 98.7%, respectively. In contrast, the NSP5 gene showed higher conservation, displaying 94.8–98.7% nucleotide identity and 97.5–100% amino acid identity relative to H1 genotype references. Phylogenetic trees were reconstructed based on the complete coding sequences of the NSP3 and NSP5 genes from the HN240916-P5 strain, alongside representative T- and H-genotype reference sequences obtained from GenBank. Specifically, the NSP3 gene clustered within the T1 lineage, showing the closest evolutionary relationship with the human strain RVA/Human/LL4260/China/T1 (Figure 2C). Concurrently, the NSP5 gene grouped into the H1 lineage and was most closely related to the human strain RVA/Human-wt/CHN/E931/2008/G9P[6] (Figure 2D). Phylogenetic analysis revealed that the VP1, VP2, VP3, VP6, NSP1, NSP2, and NSP4 genes of HN240916-P5 strain were related to lineages R1, C1, M1, I5, A8, N1, and E1, respectively (Figure 2E). In addition, all the seven gene segments of HN240916-P5 strain clustered together with porcine strains identified in China and other countries.

### 3.5 Sequence divergence of VP7 and VP4 for neutralizing epitopes

Alterations in amino acid sequences within antigenic epitopes of the RV VP7 trimers and VP4 multimers may compromise vaccine efficacy. Trypsin cleavage of VP4 generates VP8 (26 kDa) and VP5 (60 kDa) fragments. VP5 contains five neutralizing antigenic domains (5-1 to 5-5), while VP8 encompasses four domains (8-1 to 8-4) and VP7 epitopes are organized into two domains (7-1, 7-2) (28, 29). Multiple putative trypsin cleavage sites were identified in VP4 at positions 231, 241, 247, 467 and 582, characterized by conserved arginine residues. Lysine at position 258 was additionally proposed as a potential cleavage site (19). Notably, HN240916-P5 strain retained all conserved residues at these loci (data not shown).

**Figure 3:**
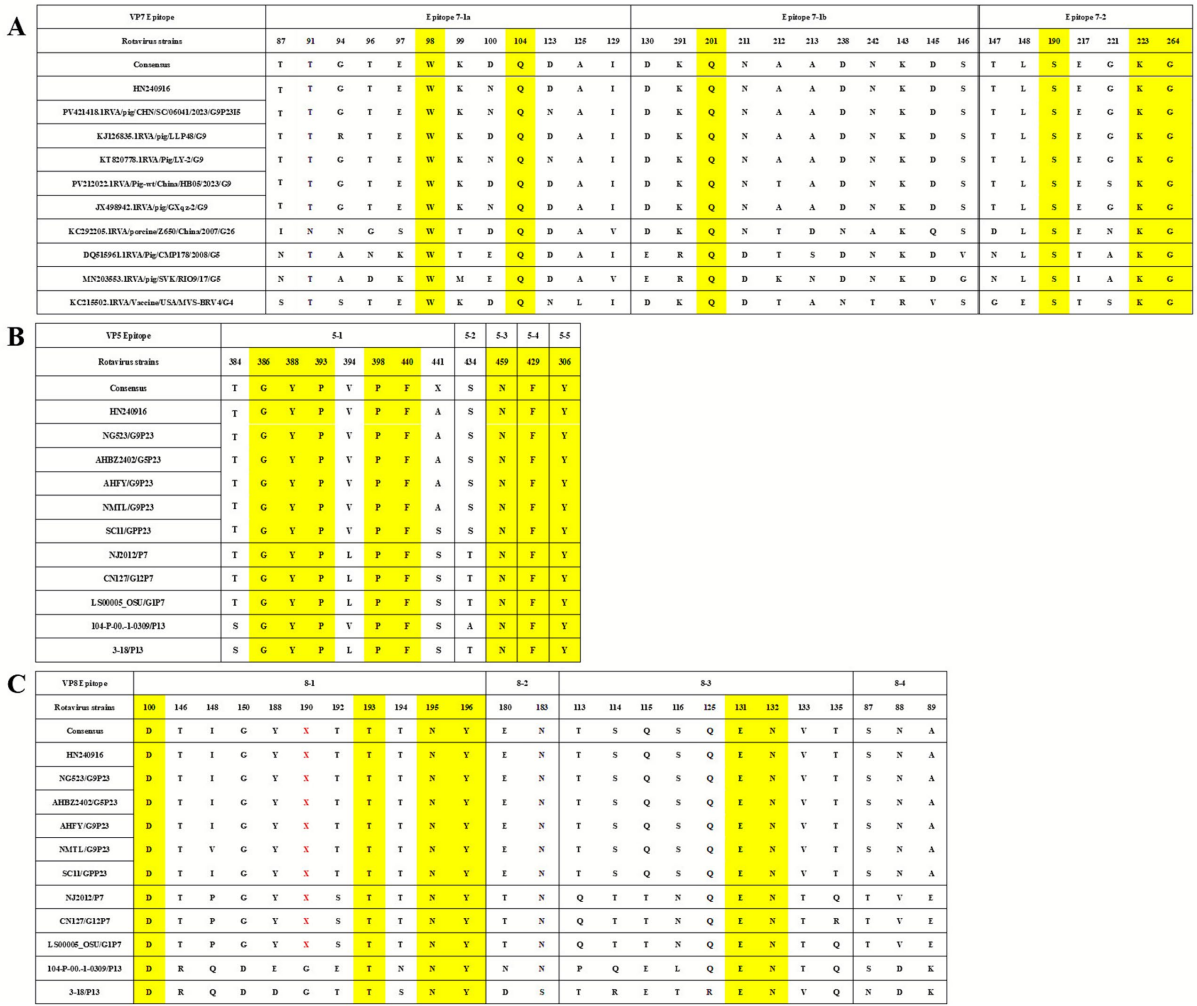
Comparison of the VP7 and VP4 (VP5* and VP8*) antigenic epitopes between HN240916-P5 strain and other reference PoRV strains. **(A)** Neutralizing epitopes on the VP7 protein. **(B)** Neutralizing epitopes on the VP5* protein. **(C)** Neutralizing epitopes on the VP8* protein. The yellow loci represent the same residues. The red X letters represent the amino acid deletion.

Comparison of the neutralizing epitope regions (7-1 and 7-2) in VP7 of the HN240916-P5 with HB05 (PV212022.1) and other multiple PoRV strains from GenBank revealed conserved amino acid residues in 7-1 (98W, 104Q, and 201Q) and 7-2 (190S, 223E and 264G) (Figure 3A). However, strain-specific variations between HN240916-P5 and other selected G9 isolates were detected, including RVA/pig/CHN/SC/06041/2023/G9P23I5 at position 123, LLP48 at positions 94 and 100, LY-2 at position 123, and RVA/Pig-wt/China/HB05/2023/G9 with three site variations at positions 100, 212 and 221 (Figure 3A). A specific comparison of the neutralizing epitope regions in VP4 of the HN240916-P5 strain with AHFY2022 (OQ979283.1) and other multiple PoRV strains from GenBank revealed that VP5 epitopes contained eight conserved amino acid residues (306Y, 386G, 388Y, 393P, 398P, 429F, 440F, and 459N) and four variable sites (384, 394, 441, and 434) (Figure 3B), whereas only six amino acids were conserved across 25 positions in VP8 epitopes, four in 8-1 (100D, 193T, 195N, and 196Y) and two in 8-3 (131E and 132N) (Figure 3C).

## 4 Discussion

RV, a leading cause of acute gastroenteritis in human infants and young livestock, manifests with emesis, watery diarrhea, and dehydration. Originally isolated from diarrheic calves in 1969, RV has since been detected in humans, swine, and other species (30–32). Infections in adult animals and humans typically present as subclinical. RV claims about 450,000 lives per year, disproportionately affecting Asian and African resource-limited settings (33, 34). PoRV circulates endemically worldwide, inflicting substantial economic losses on swine production systems. In the present study, we isolated a PoRV strain, HN240916, from a large-scale pig farm experiencing a severe diarrhea outbreak. The detection of PoRV as the sole viral agent in diarrheic piglets suggests that it may be the primary etiological agent responsible for this outbreak. However, the pathogenicity of the HN240916 strain requires further investigation. The pig farm from which samples were obtained is located in Nanyang City, Henan Province, which constitutes the geographical center of China, a position of potential epidemiological relevance. It is also one of the regions with the densest concentration of pig farming. The virus strains prevalent in this area are highly representative. At the same time, the region is currently underrepresented in systematic PoRV surveillance. The present report helps fill this critical geographic gap and expands the understanding of the epidemiological distribution of PoRV.

PoRV isolation and cell culture propagation enable critical investigation of its pathogenicity in piglets. The virus was initially adapted for cultivation in primary porcine kidney cells pretreated with trypsin. Bohl et al. (35) successfully isolated RV using MA104 cells in 1984 for the first time. Subsequent studies have also reported the successful isolation of RV using various cell lines, including Caco-2, Vero, MARC-145, and IRBS II (36). In practice, isolating RV remains challenging due to the complexity of clinical samples. In this study, the MA104 cell line was used for the isolation of PoRV. By optimizing the virus isolation method, especially the trypsin concentration for viral activation during pretreatment, a strain of PoRV was successfully isolated and identified as PoRV group A via HTS, designated as HN240916. Isolation of RVs is generally challenging due to their dependence on trypsin-mediated activation for efficient infection in MA104 cells. Trypsin cleaves the VP4 protein into VP5 and VP8, which facilitate viral attachment and entry in cooperation with VP7. A previous study has shown that proteolytic processing enhances viral infectivity by promoting VP4 conformational rearrangement (37). These findings suggest that fine-tuning protease activation conditions is critical for efficient PoRV isolation and provide new insights into the mechanisms underlying RV entry and replication. Following three blind passages in MA104 cells, stable CPE was manifested and characterized by cell elongation and detachment, consistent with previous reports (38, 39). The virus was further identified by PCR, IFA, and TEM. These results conclusively demonstrate the successful isolation of the novel PoRV HN240916 strain.

According to the classification method of RV, the genotype of the HN240916 strain was determined as G9-P[23]-I5-R1-C1-M1-A8-N1-T1-E1-H1. It was found to be identical to the previously identified Chinese PoRV G9P[23] strains, such as SC11 and HB05, which have the same genotype (40, 41). In recent years, the detection rate of G9 PoRV has been increasing, and G9 RV are considered to be emerging genotypes in pigs and humans worldwide (42–45). G9 RV strains are typically associated with P[7], P[13], P[19], and P[23]. In this study, a new G9 strain was isolated from MA104 cells and combined with P[23] to form the G9P[23] genotype. However, in recent years, uncommon G and P genotypes of PoRV have been detected in different regions of the world, increasing the difficulty in preventing and controlling the disease (46, 47).

Genome-wide phylogenetic analysis revealed a distinct pattern in the genetic origins of the HN240916 strain. The VP1, VP2, VP3, VP4, VP6, VP7, NSP1, NSP2 and NSP4 gene segments form a monophyletic cluster with PoRV strains. In contrast, the NSP3 and NSP5 gene segments exhibited an evolutionary branch with human RV lineages. Together, this genotype constellation provides definitive genomic evidence that HN240916 is a porcine-human reassortment virus. This finding aligns with several reports of similar interspecies reassortment, indicating that it is a common phenomenon in the evolution of RVs (40, 41, 48). The widely existing of these reassortments reinforces the critical importance of continuous and integrated surveillance of both porcine and human RVs to monitor the emergence of novel strains. In addition, NSP3 and NSP5 are involved in viral replication and assembly, and their potential human origin may confer replication advantages or contribute to host adaptation. This remains a subject for future experimental research. Ultimately, the identification of HN240916 underscores the dynamic nature of RV genome evolution and highlights cross-species gene exchange as an important driver of RV diversity.

A detailed analysis of the amino acid sequences of the VP7 and VP4 genes of the HN240916 strain has revealed the occurrence of certain amino acid mutations. Therefore, further studies on HN240916 strain are warranted. VP7 and VP4 serve as the major antigens capable of eliciting neutralizing antibodies against RV infection. Compared to porcine G9 and P[23] genotype strains, the HN240916 strain exhibits significant divergence at both the nucleotide and amino acid levels, which may potentially compromise the efficacy of existing vaccines. This highlights the importance of continuous molecular monitoring of circulating strains in China, particularly G9 variants with zoonotic potential. Such surveillance is essential to support the rational design of multivalent vaccines that reflect the prevailing genetic landscape.

## 5 Conclusion

In summary, the study reports the isolation and whole-genome characterization of a PoRV strain in Henan Province, designated as HN240916, which was successfully propagated in MA104 cells. The biological characteristics of the strain were identified using HTS, IFA, and TEM. Whole-genome sequencing revealed that strain HN240916 possesses a G9-P[23]-I5-R1-C1-M1-A8-N1-T1-E1-H1 genotype constellation. These findings expand the molecular epidemiological landscape of PoRV in an underrepresented region, provide evidence of ongoing interspecies reassortment, and underscore the importance of continuous RV surveillance. They also offer valuable insights into the genetic evolution of PoRV and may inform future control strategies and vaccine strain selection.

## Data Availability

The datasets presented in this study can be found in online repositories. The names of the repository/repositories and accession number(s) can be found in the article/supplementary material.
